# sFasL-mediated induction of neutrophil activation in patients with type 2 diabetes mellitus

**DOI:** 10.1371/journal.pone.0201087

**Published:** 2018-07-19

**Authors:** Sona Margaryan, Agata Witkowicz, Arsen Arakelyan, Anna Partyka, Lidia Karabon, Gayane Manukyan

**Affiliations:** 1 Russian-Armenian University, Yerevan, Armenia; 2 Laboratory of Molecular and Cellular Immunology, Institute of Molecular Biology, Yerevan, Armenia; 3 Department of Experimental Therapy, L. Hirszfeld Institute of Immunology and Experimental Therapy, Polish Academy of Sciences, Wroclaw, Poland; 4 Bioinformatics Group, Institute of Molecular Biology, Yerevan, Armenia; Universite Paris-Sud, FRANCE

## Abstract

Fas/Fas ligand system was shown to be related to insulin resistance and type 2 diabetes mellitus (T2DM). However, the role of soluble Fas ligand (sFasL) in functioning of immune cells in type 2 diabetes mellitus (T2DM) has not been studied yet. The aim of the present study was to determine in vitro effects of sFasL on neutrophil activation and apoptosis. We demonstrate here that sFasL exhibited proinflammatory effect and induced mRNA levels of caspase-1, NF-κB, IL-1β and CD18 expression. At the same time, sFasL induced reactive oxygen species (ROS) production. Activation of caspase-1 activity abolished sFasL-dependent apoptosis, and suppressed Fas expression and mRNA levels of caspase-3 in neutrophils from T2DM patients. Collectively, our findings identify a novel proinflammatory role of sFasL in T2DM neutrophils that is dependent of caspase activity. Thus, sFasL enhances inflammatory response of neutrophils from T2DM patients without increasing apoptosis suggesting its triggering role in T2DM inflammation.

## Introduction

Type 2 diabetes mellitus (T2DM) is characterized by chronic insulin resistance and a progressive decline in β-cell function. It is recognized that obesity and low-grade chronic inflammation are risk factors for T2DM [1–3]. A number of animal models have demonstrated loss of β-cell mass. It was proposed that increased apoptosis rather than decreased neogenesis or replication may be the main mechanism leading to reduced β-cell mass [4]. The molecular mechanisms lying behind deregulated apoptosis in the human β-cell are not fully clear. Several reports indicate that high glucose concentrations and free fatty acids among others are factors of altered β-cell proliferation, apoptosis and function [5]. Recently, Fas/FasL pathway was shown to be related to insulin resistance and type 2 diabetes mellitus (T2DM) [6]. It was shown that increased Fas expression contributes to impaired insulin sensitivity and adipose tissue dysfunction in obesity, while weight loss reduces its expression. Moreover, adipocyte-specific deletion of Fas improved insulin sensitivity and relieved adipose tissue inflammation in high fat diet-fed mice [7, 8].

FasL (CD178) is a type II homotrimeric transmembrane protein that belongs to the TNF gene family. It is well known that FasL and Fas receptor interaction play an important role in the control of immune cell homeostasis [9]. The diverse activities of FasL arise from functional differences in membrane-bound (mFasL) and soluble (sFasL) forms of the molecule. The overall function of FasL is the result of the separate contributions of mFasL and sFasL, which have opposing functions in apoptosis and inflammation. Soluble FasL can be released from the cell surface by metalloproteinase-mediated cleavage of membrane-bound FasL [10]. Upon stimulation by mFasL, cells expressing Fas receptor causes apoptosis by recruiting different adaptor proteins which cleavage of procaspase-8, the most upstream caspase in the Fas apoptotic pathway. Molecular details underlying proinflammatory signaling induced by sFasL are not completely understood. It is believed that sFasL does not efficiently induce apoptosis; it blocks mFasL-mediated apoptosis and induce proinflammatory and chemotactic activity of immune cells [11, 12]. The studies evaluating the correlation between diabetic complications and apoptosis have shown that Fas/FasL axis plays role in development of diabetic retinopathy, nephropathy and neuropathy [13–15]. Conflicting results were obtained regarding serum levels of FasL. Several studies, including ours (unpublished), have detected increased levels of sFasL in the serum of T2DM patients [15, 16], while another demonstrated its decreased levels compared to healthy subjects [17]. The aim of the present study was to define *in vitro* the biological effects of sFasL on neutrophil activation and apoptosis.

## Material and methods

### Patients

Peripheral blood samples of 17 subjects (mean age 45.4 ± 10.1) diagnosed with T2DM were recruited from the “Surb Astvatsamayr” Medical Center (Yerevan, Armenia). Patients met the criteria established by an expert committee on the diagnosis and classification of diabetes mellitus: i) random venous plasma glucose ≥11.1mmol/L, ii) fasting plasma glucose ≥7mmol/L, iii) laboratory HbA1c >48mmol/mol (6.5%). All the patients with T2DM were treated with diet therapy and oral hypoglycemic agents. No patients received insulin therapy and were without any diabetic complications. Patient characteristics are summarized in Table 1. A total of 15 healthy controls (mean age 34.6 ± 5.3) with similar ethnic background, neither suffering from obesity, T2DM, nor from other medical disorders (including acute infection and metabolic diseases), were recruited as well. Healthy controls were not perfectly age-matched with the patients which remains a limitation of our study. The study was approved by the Ethical Committee of the Institute of Molecular Biology of the National Academy of Sciences of the Republic of Armenia (IRB IORG0003427) and all participants gave written informed consent.

**Table 1:** Clinical and demographic data of T2DM patients.

Male/Female	7/10
Age (years)	45.4 ± 10.1
Duration of diabetes (years)	9.3 ± 5.8
BMI (kg/m^2^)	27.3 ± 2.4
Fasting glucose (mmol/L)	10.4 ± 3.2
HbA_1C_ (%)	7.2 ± 0.6
LDL cholesterol (mmol/L)	2.9 ± 0.33
HDL cholesterol (mmol/L)	1.1 ± 0.25
Treatment gliclazide/metformin containing medicines	5/12

### Sampling

Peripheral blood samples were collected in tubes containing EDTA and processed within 2 hour after collection. Neutrophils were isolated by density centrifugation using the Histopaque-1077 gradient technique (Sigma, St. Louis, MO) according to the manufacturer’s protocol. Contaminating erythrocytes were removed from the granulocyte fraction by brief hypotonic lysis. Granulocyte purity, as determined by counting of cytospin preparations stained with May-Grundwald Giemsa, was always greater than 95%. Viability of neutrophils, assessed by trypan blue exclusion, was greater than 95% immediately after purification.

Neutrophils (5x10^6^ cells/ml) were cultured at 37°C in RPMI 1640 (Sigma, St. Louis, MO) supplemented with 10% fetal calf serum, 2 mmol/L L-glutamine and 1 mM sodium pyruvate in the absence or presence of 150 ng/ml FasL (Biolegend, UK) in a total volume of 1 ml for 3 hours at 37°C. After stimulation or directly after isolation, neutrophils were washed once with cold PBS, and stored in 150 μl RNAlater (Sigma, St. Louis, MO) at -20°C until use.

### RNA extraction and RT-PCR

Total RNA was extracted from frozen cells using the Qiagen RNeasy Mini Kit (Qiagen, Germany) according to the manufacturer’s protocol. The RNA concentration was determined spectrophotometrically. Quality of the RNA was checked spectrophotometrically and also by visualization of the 28S:18S ribosomal RNA ratio on a 2% agarose gel. Immediately after isolation the 500 ng of total RNA was reverse transcribed with the High Capacity RNA–to-cDNA kit (AppliedBiosystems, Thermo Fisher Baltics, Vilnus, LT)).

The mRNA levels of human IL-1β, NF-kB, caspase-1, Ncf-1, catalase, caspase-3, Bax, and Bcl-2 mRNA were determined using the following assays:Hs01555410_m1, Hs01042014_m1, Hs00354836_m1, Hs00165362_m1, Hs00156308_m1, Hs00234387_m1, Hs00180269_m1, Hs00608023_m1, respectively (AppliedBiosystems, Foster City CA, USA). As reference gene β2M was used with applying of Pre-developed TaqMan Assay Reagents Human β2M. All of the samples were assayed in duplicate on the ViiA 7 Real Time PCR System (AppliedBiosystems, USA). The results were calculated according to the ΔCt method [18].

### *In vitro* stimulation of whole blood with FasL

To investigate the *in vitro* effect of sFasL, aliquots of whole blood were diluted 1:10 with RPMI-1640 medium. The blood was distributed in 24-well plates and stimulated with 150 ng/ml sFasL for 3 hours at 37°C. To determine the dose-effect curves for sFasL, kinetic studies were performed (data not shown). Following culturing, the supernatants were collected and frozen at -70°C until determination of cytokines concentrations, and the cells were taken for the cytometric analyses.

### Antibodies used for flow cytometry

APC/Cy7 anti-human CD15 (Biolegend, UK) was used to discriminate neutrophils in whole blood staining. FITC anti-human CD62L, PE anti-human CD18, and APC anti-human CD11b (Biolegend, UK) were used to detect surface expression of adhesion molecules. Irrelevant antibodies of the same isotypes were used as negative controls.

### Flow cytometric analysis

At the end of stimulations, cells were washed and harvested. Afterwards, the cells were incubated with fluorescent mAb toward CD14, CD16, CD11b, CD18, and CD62L for 30 minutes. Labeled cells, after further washing, were resuspended in PBS supplemented with 1% BSA. Antigen expression was analyzed on a Partec CyFlow Space (Partec, Germany). 10 000 events were collected from each sample. The neutrophil population in peripheral blood was distinguished by side scatter, CD16^bright^ positivity and CD14 negativity. The monocyte population was determined as CD14^++^/CD16^-^ cells. Gating and determination the median fluorescence intensity (MFI) were done by FlowJo software (FlowJo, Ashland, OR, USA).

### Oxidative burst assay

Neutrophil oxidative stress capacity was analyzed using dihydrorhodamine 123 (DHR-123) (Sigma-Aldrich), a nonfluorescent compound which accumulates in mitochondria and is converted to the highly fluorescent rhodamine-123 by the action of oxidative stress. Briefly, the tubes with whole blood were prelabeled with CD15 and incubated with 20 μM DHR-123 in the dark at 37°C for 20 min. Then 150 ng/ml sFasL was added and the cell suspensions were incubated in the dark at 37°C for further 20 min. For the negative control, whole blood cells were incubated with DMSO. After lysis of erythrocytes, remaining cells were resuspended in PBS, placed on ice and analyzed with flow cytometry similarly to the analysis of surface antigens.

### Annexin V binding assay

After culturing of whole blood from T2DM patients and healthy controls, the cells were lysed to remove erythrocytes, washed in annexin-binding buffer, stained with 5 μL of Annexin V–FITC conjugate for 20 minutes, followed by staining with 1 μg/mL of propidium iodide (PI), and analyzed by flow cytometry.

### IL-1β and IL-8 protein quantification by ELISA

Cytokine release in supernatants was measured with specific immunoassays according to the manufacturer's instructions. Cytokine production was analyzed using Human IL-1β and IL-8 ELISA MAX Deluxe kits (Biolegend, UK). The samples were read at 450 nm in a 96-well plate reader (HumaReader HS, Human Diagnostics Worldwide, Germany). Results were calibrated with serial dilutions of known quantities of recombinant cytokines.

### Statistics

Statistical analyses were carried out using the Statsoft Statistica package (http://www.statsoft.com). All values are given as means ± standard errors of the means. Normal distribution was checked visually from distributions and with Shapiro-Wilk's W test. For continuous variables, groups were compared using the paired Student’s t-test. *P* values ≤ 0.05 were considered as significant.

## Results

### Baseline mRNA levels of studied molecules in T2DM neutrophils are unchanged

**Fig 1:**
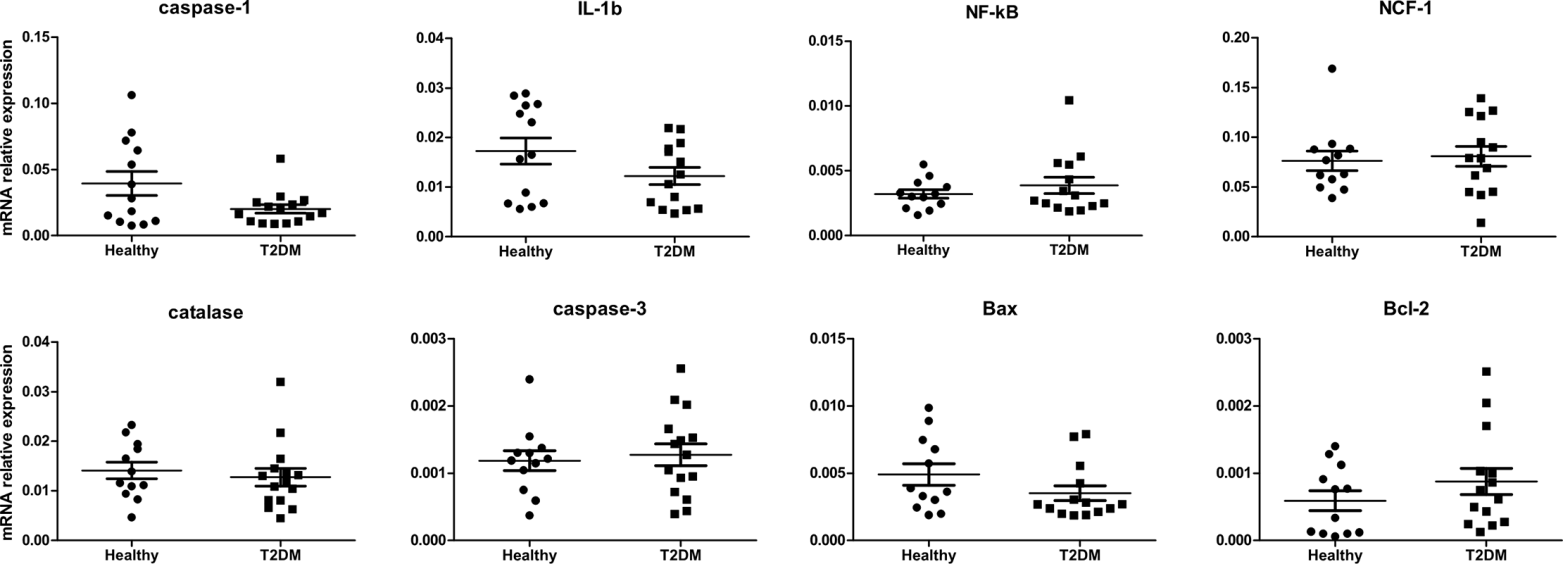
Baseline mRNA levels of candidate genes in neutrophils from T2DM patients (T2DM) and healthy controls (healthy) measured by quantitative RT-PCR. All gene expressions are relative to β2M. Data are represented as scatterplots. Error bars are means ± SEM for each group.

Baseline gene expression was evaluated in peripheral blood neutrophils from healthy subjects and T2DM patients using qRT-PCR. Freshly isolated neutrophils from T2DM patients did not exhibit significant differences in mRNA expression of investigated genes (caspase-1, IL-1β, NF-κB, Ncf-1, catalase, caspase-3, Bax, and Bcl-2) as compared to healthy subject (Fig 1).

### Induction of sFasL-mediated levels of pro-inflammatory mediators in T2DM

**Fig 2:**
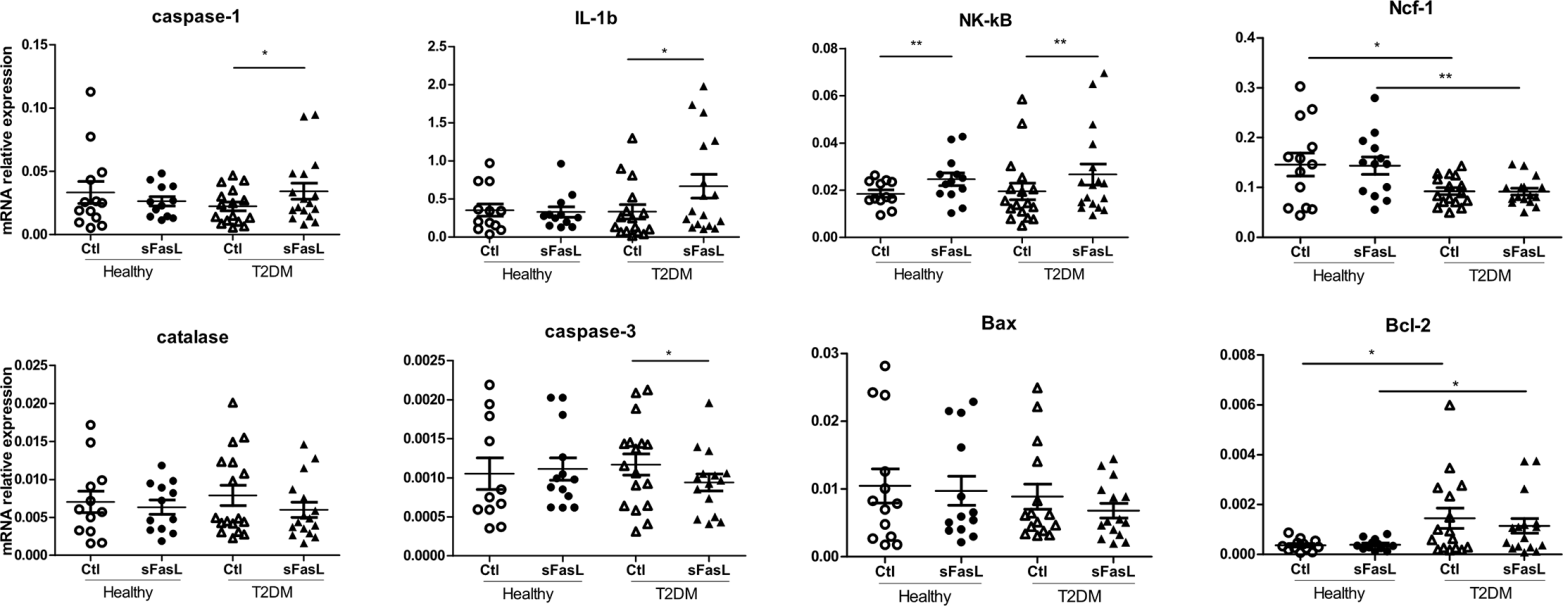
Relative mRNA levels of candidate genes in neutrophils T2DM patients (T2DM) and healthy controls (healthy) after 3-hour culture with media alone as a control (Ctl) or sFasL (150ng/ml) measured by quantitative RT-PCR. All gene expressions are relative to β2M. Data are represented as scatterplots. Error bars are means ± SEM for each group (*P<0.05, **P<0.01).

We have investigated the possibility whether sFasL is able to activate proinflammatory signaling pathways in neutrophils from T2DM patients. As illustrated in Fig 2, sFasL significantly increased mRNA levels of NF-κB (*P*<0.01), IL-1β (*P*<0.05) and caspase-1 (*P*<0.05) in neutrophils from T2DM patients, while healthy cells were less responsive to the inducer. In response to sFasL, only mRNA of NF-κB increased significantly in healthy cells (*P<*0.05), what occurred in the absence of changes in the mRNA levels of IL-1β and caspase-1 (Fig 2).

**Fig 3:**
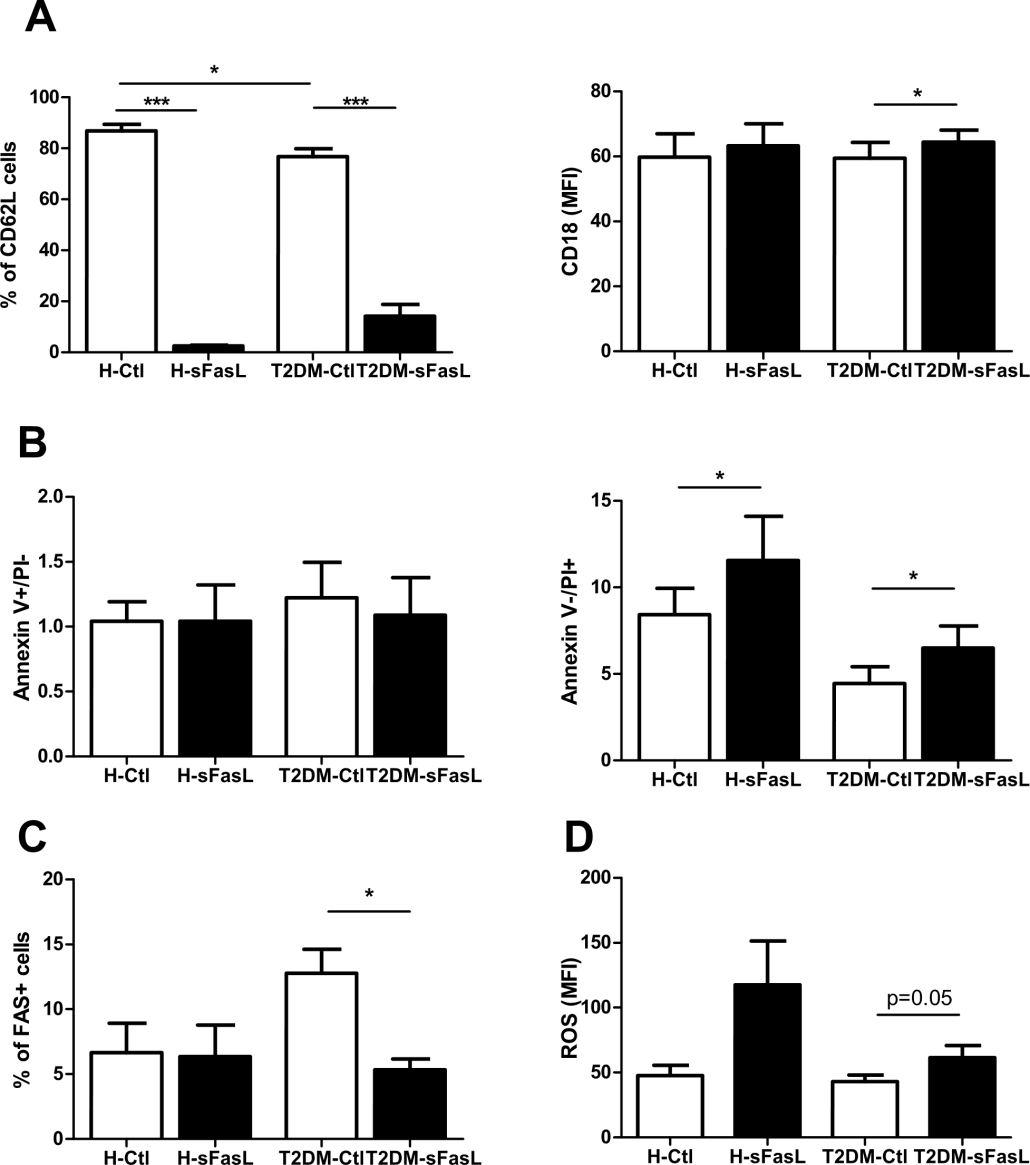
The influence of sFasL on neutrophils from T2DM patients (T2DM) and healthy controls (H) after 3-hour culture with media alone as a control (Ctl) or sFasL (150ng/ml) measured by flow cytometry: **A)** Percentage of CD62L and expression levels of CD18; **B)** Apoptotic rates of circulating neutrophils measured using Annexin V and Propidium iodide (PI); **C)** Percentage of Fas positive cells; **D)** Median fluorescence intensity (MFI) of ROS measured using dihydrorhodamine 123.

The expression of CD62L (L selectin) and CD18 (integrin beta-2) was analyzed by flow cytometry on the surface of resting and treated with sFasL neutrophils. sFasL exposure dramatically reduced the percentage of CD62L expressing cells from both T2DM patients and healthy controls (*P<0*.001). The percentage of CD62L positive cells were also reduced in cells from T2DM patients if compare with those from healthy individuals (for untreated cells P<0.05, for sFasL-treated P = 0.06) (Fig 3A). The intensity of CD18 expression was slightly up-regulated only in T2DM group after exposure of the cells with sFasL (*P<*0.05) (Fig 3A).

### Effect of sFasL on cell apoptosis and the expression of apoptosis-related gene expression

Spontaneous apoptotic rates of circulating neutrophils were not different in both investigated groups (Fig 1). It is noteworthy that 3h incubation with sFasL did not affect neutrophil apoptosis evaluated by the percentage of neutrophils displaying the typical morphological features of apoptosis (data not shown) and flow cytometric analysis of Annexin V-FITC binding. Cell survival evaluated as cell viability was slightly decreased in the group of diabetic patients. sFasL treatment of the cells has led to the increased number of dead cells in both investigated groups (Fig 3B).

Besides, we initially analyzed cell surface expression of Fas by FACS method. Fas transmit apoptotic signals into susceptible cells, therefore we tested whether the Fas receptor expression on the surface of neutrophils corresponds to the cell death upon ligation with sFasL. Fas receptors were constitutively expressed and were not significantly modulated by the treatment with sFasL on neutrophils from healthy subjects, while in T2DM patients the percentage of Fas expressing cells was lower after the induction (*P<*0.05) (Fig 3C). However, when we analyzed the intensity of Fas expression, it was not different neither in cultivated nor in resting cells in both investigated groups (data not shown).

In parallel, we have analyzed mRNA expression levels of caspase-3, Bax, and Bcl-2. Constitutive transcriptional levels of these genes were not different in studied healthy and diseased groups (Fig 1). sFasL exposure resulted in down-regulated levels of caspase-3 in neutrophils from T2DM patients (P<0.05), which occurred in the absence of changes in the levels of pro-apoptotic Bax and anti-apoptotic Bcl-2. In neutrophils from T2DM patients, Bcl-2 mRNA expression was significantly increased compared to neutrophils from healthy controls in both stimulated with sFasL and unstimulated cultures (Fig 2).

### Reactive oxygen species production by sFasL-induced whole blood neutrophils

Having observed the influence of sFasL on inflammation-related gene expression, we aimed to measure the production of reactive oxygen species (ROS) as well as the levels of Ncf-1 and catalase transcripts. The production of ROS at basal level was 2-fold higher in neutrophils from T2DM compared to controls, albeit the difference was not significant (data is not shown). sFasL-treated cells from T2DM patients demonstrated the tendency (P = 0.05) towards increased production of ROS compared to the control cells (Fig 3D). In concordance with ROS production, the catalase mRNAs in the cells from T2DM were slightly reduced after sFasL treatment (P = 0.05). In contrast, Ncf-1 mRNAs were not influenced by the inducer, however, treated and untreated neutrophils from healthy subjects demonstrated higher expression of Ncf-1 then in diseases affected cells (P <0.05 and P<0.01, respectively) (Fig 2).

### Increased production of IL-8 following sFasL exposure by whole blood cells in T2DM

Having established that exposure of human neutrophils to sFasL induced upregulation of IL-1β at the mRNA level, we examined whether this mRNA was translated into protein. Unexpectedly, protein levels of IL-1β were not affected by the sFasL treatment (*P*>0.05). The reasons for the delay in IL-1β mRNA translation are unclear. The impaired translation of IL-1β could be due to the lack of cultivation time or translation defects in the cells from T2DM patients.

**Fig 4:**
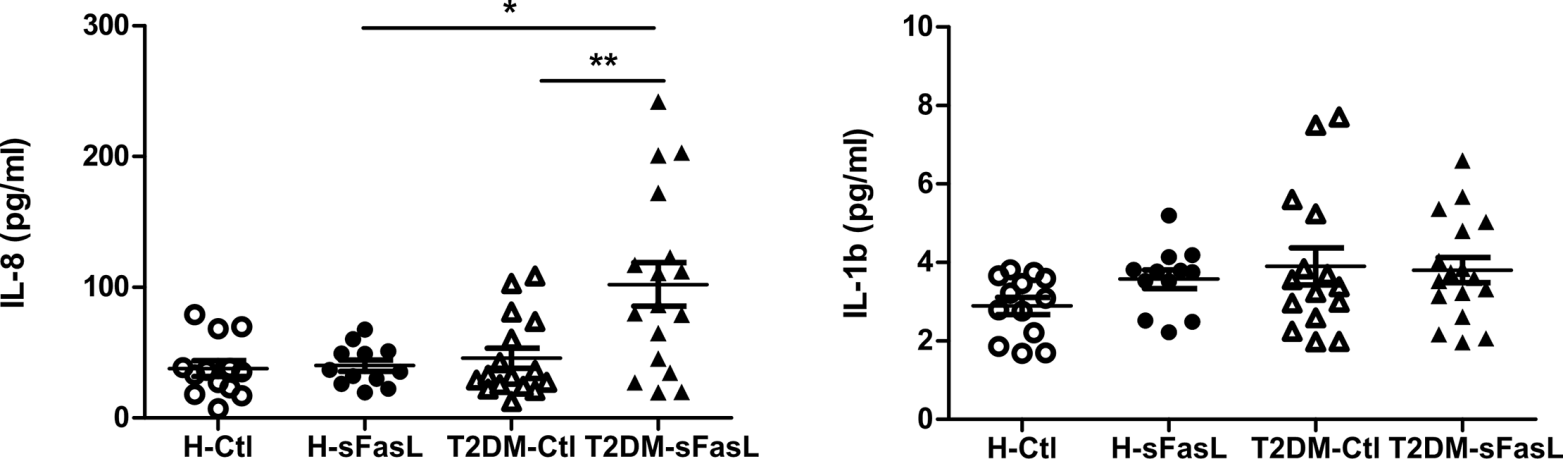
Secreted levels of cytokines IL-1β and IL-8 from T2DM patients (T2DM) and healthy controls (H) after 3-hour culture of isolated neutrophils with media alone as a control (Ctl) or sFasL (150ng/ml) measured by ELISA. Data are represented as scatterplots. Error bars are means ± SEM for each group (*P<0.05, **P<0.01).

As flow cytometry data have shown significant impact of sFasL on selectin and integrin levels, we analyzed whether IL-8 protein levels correspond to the altered expression of these molecules. In T2DM, sFasL stimulation resulted in significant increased production of IL-8 compared to control cultivation (*P*<0.01), and compared to sFasL-induced cultivation in healthy group (*P*<0.05) (Fig 4).

## Discussion

T2DM is an inflammatory disease with a chronic low-grade activation of the innate immune system [19, 20]. Growing evidences suggest neutrophils as active players in obesity-induced inflammation and insulin resistance [21]. Moreover, increased neutrophil count was shown to be associated with insulin resistance, β-cell dysfunction [22], hyperglycemia and what is more it is proposed as a predictor of the incidence of T2DM [23]. Our main goal was to define the role of sFasL in neutrophil activation in T2DM patients.

sFasL exhibited proinflammatory effect and induced increased activation of circulating neutrophils from T2DM patients. Following stimulation with sFasL, neutrophils release a proinflammatory cytokine IL-1β, which synthesized as a precursor after NF-κB-mediated transcriptional induction. Secretion of bioactive IL-1β depends on activation of caspase-1 by a multi-protein complex termed the inflammasome [24, 25]. Despite of increased mRNA levels of NF-κB after sFasL exposure in both groups, our findings show that the mRNA expression of IL-1β and caspase-1 was upregulated only in neutrophils from the patients. This fact may suggest the involvement of inflammasome assembly in the process of neutrophils activation in T2DM patients in the presence of sFasL. The activation of the NLRP3 inflammasome was shown to be positively associated with the prevalence of T2DM [26, 27]. Despite, the constitutive expression of inflammation-related genes was not different from those of healthy subjects, increased ability of neutrophils to activate inflammasome-related molecules in the presence of sFasL may represent an important pathogenic mechanism responsible for chronic inflammation in T2DM.

Activation of proinflammatory pathways in neutrophils exposed to sFasL was further confirmed by the increased ROS production, expression of CD18 and decreased number of CD62L+ cells (shedding effect). ROS play a crucial role in the progression of inflammatory disorders and its increased production may contribute to alterations of insulin/insulin receptor substrate signaling pathways leading to insulin resistance and inflammatory settings [28]. Consistent with previous observations of changes in ROS production [29], slightly increased sFasL-induced ROS levels were detected. Fas ligation is well known to induce a cell-intrinsic apoptotic pathway resulting in mitochondrial damage. In line with our data, depletion of hepatic Fas expression enhanced mitochondrial respiration and the abundance of respiratory complexes [30]. Oxidative stress activates several inflammatory pathways such as inflammasome assembly, increased expression of adhesion molecules and cytokines as well as alters phospholipase activity, activate MAP kinase, STAT and TLR signaling pathways [31]. Together, these data demonstrate that redox imbalance and increased proinflammatory activity of T2DM neutrophils in response to sFasL may contribute to the maintenance of the inflammatory processes in T2DM.

It is well accepted that apoptosis is a cause of a relative β-cell deficiency in T2DM [4]. It was shown that β-cells exposed to high glucose levels secrete IL-1β, thus activating NF-κB and Fas signaling and consequently triggering apoptosis [32]. Being critical mediator of β-cell death in type 1 diabetes [33], NF-κB/IL-1β signaling pathway was implicated in β-cell death in both types of diabetes [34]. The factors such as hypoxia [35, 36], nitric oxide [37] stimulate the release of soluble FasL into the circulation. In another study, failing myocytes were proposed to be responsible for the increased circulating levels of sFasL [38]. Whether pancreatic β-cell damage may contribute to the release of sFasL into the circulation is unknown. This fact has not been proven yet and data available on the serum levels of sFasL point to the increased sFasL levels in T2DM patients with hypertension, vascular complications [39], and neuropathy [15]. Neutrophils in both healthy and T2DM groups were resistant to the sFasL-mediated apoptosis which further confirms proinflammatory properties of this molecule. Moreover, sFasL-induced activation of neutrophils was associated with decreased caspase-3 mRNA levels and the percentage of Fas expressed cells. Downregulation of Fas may be involved in antiapoptotic action induced by sFasL and trigger signaling pathways which cause activation of survival factors [35]. It is well known that caspase 8 (FLICE)-like inhibitory protein (cFLIP) suppresses apoptosis by forming heterodimers with pro-caspase 8 to inhibit its activation, and divert Fas-mediated death signals into those for cell proliferation [40, 41]. Moreover, a pivotal role cFLIP was illustrated by showing its capability to protect β cells from glucose-induced apoptosis, restor β cell proliferation, and improve β cell function [42]. The detailed molecular mechanism of regulatory role cFLIP for survival signaling from sFasL in neutrophils needs to be addressed further in future studies.

sFasL-induced upregulation of IL-8 production observed here is highly consistent with previous findings showing chemoattractant properties of sFasL at doses insufficient to induce apoptosis [43]. Additional support to our data was provided earlier by *Hatanaka et al*, who demonstrated excessive basal and LPS-induced release of cytokines by neutrophils, including IL-8 [44]. In tissues, activated neutrophils may lead to the tissue injury as it was shown recently by *Talukdar et al*, who showed increased neutrophil content in adipose tissue and liver in high fat diet obese mice. It was suggested that neutrophils play essential role in the onset of the disease as neutrophil recruitment into adipose tissue might be the initial event of T2DM inflammation [21, 45]. Increased production of IL-8 by neutrophils may create a gradient between adipose tissue and circulation, promoting chemotaxis of macrophages and other immune cells. Besides, IL-8 shifts the balance between metalloproteinases and their inhibitors in favor of the former [46], which may further contribute to the tissue damage.

Our study was not powered to assess the impact of different therapy (gliclazide/metformin) on activation status of neutrophils, which should be addressed in a larger cohort of T2DM patients.

In conclusion, sFasL has potent proinflammatory and chemotactic effect on neutrophils in T2DM. Our results provide new mechanistic insights into understanding the molecular activation of the neutrophils mediated by sFasL which may ultimately contribute to the sustaining inflammation in T2DM. Our data raise new questions on the involvement of sFasL in the events leading to increased concentrations of active IL-1β and/or IL-8 in T2DM.
